# Complete Genome Sequence of the Largest Known Flavi-Like Virus, *Diaphorina citri flavi-like virus*, a Novel Virus of the Asian Citrus Psyllid, *Diaphorina citri*

**DOI:** 10.1128/genomeA.00946-16

**Published:** 2016-09-08

**Authors:** Emilyn E. Matsumura, Luca Nerva, Jared C. Nigg, Bryce W. Falk, Shahideh Nouri

**Affiliations:** a Department of Plant Pathology, University of California, Davis, California, USA; b Instituto de Biociências de Botucatu, Universidade Estadual Paulista, Botucatu, SP, Brazil; c Laboratório de Biotecnologia, Centro APTA Citros Sylvio Moreira, Instituto Agronômico de Campinas, Cordeirópolis, SP, Brazil; d Institute for Sustainable Plant Protection (IPSP), CNR, Turin, Italy

## Abstract

A novel flavi-like virus tentatively named *Diaphorina citri flavi-like virus* (DcFLV) was identified in field populations of *Diaphorina citri* through small RNA and transcriptome sequencing followed by reverse transcription (RT)-PCR. We report here the complete nucleotide sequence and genome organization of DcFLV, the largest flavi-like virus identified to date.

## GENOME ANNOUNCEMENT

The flavi-like viruses are a group of viruses which share genome organization and encoded proteins with viruses of the family *Flaviviridae* (1). The family *Flaviviridae* contains a diverse group of RNA viruses including numerous important pathogens from a wide range of hosts including both vertebrates and invertebrates (1). The typical *Flavivirus* genome is a positive-sense, single-stranded unsegmented RNA 9.6 to 12.3 kb in length containing a single open reading frame (ORF) (1). The ORF is translated into a single polyprotein which is subsequently cleaved into structural (CP and envelope) and nonstructural (replicase complex) proteins located at the N- and C-termini, respectively (1). Unlike the typical *Flavivirus*, the genomes of flavi-like viruses have been reported to be between 19 and 26 kb (2–6). Although the phylogenetic status of flavi-like viruses is not clear, they fall among flavivirus-jingmenvirus, pestivirus, and hepacipegivirus clades based on amino acid sequences of the NS3 (helicase) and NS5 (RdRp) proteins (4). *De novo* assembly using Trinity 2.1.1 (7) generated a contig of 27,542 nucleotides from a transcriptome library derived from *Diaphorina citri* collected in Florida. The deduced amino acid sequence displayed low amino acid sequence similarity (<40%) to the replicase proteins of flavi-like viruses using BLASTx (2–4, 6). An average coverage of 912× across the full-length of the contig was obtained by using BWA software (8). The presence of this viral sequence in *D. citri* field collected samples from Florida was confirmed by reverse transcription (RT)-PCR. The nucleotide sequences of both ends of the genomic RNA were determined by rapid amplification of cDNA ends (RACE) using the SMARTer 5′/3′ RACE system according to the manufacturer’s instructions (Clontech, Mountain View, CA). The complete genome sequence of this new putative virus tentatively named *Diaphorina citri flavi-like virus* (DcFLV) is nonpolyadenylated and is 27,724 nucleotides (nt) in length. The 5′ and 3′ untranslated regions (UTRs) are 598 and 243 nt, respectively, flanking a predicted ORF which encodes a putative polyprotein of 8,960 amino acids. The putative polyprotein contains several conserved domains including DUF612 with an unknown function (nt 1,878 to 2,088), TonB periplasmic domain (nt 1,989 to 2,089), ATP-dependent RNA helicase (nt 2,664 to 3,041), DEAD-like helicase superfamily (nt 2,738 to 2,820), Helicase superfamily C-terminal domain (nt 2,874 to 2,997), and Fts-like methyltransferase (nt 6,811 to 7,035). The putative polyprotein of DcFLV was compared in the GenBank nonredundant protein database using BLASTp with an e-value of 10^−3^, indicating that the highest identity was with *Gentian Kobu-sho-associated virus* (GKaV) (query coverage 21%; identity 39%) (GenBank accession no. BAM78287), a flavi-like virus discovered in gentian plants showing kobu-sho syndrome (3). A phylogenetic tree based on the amino acid sequences of flavivirus RdRp proteins placed DcFLV in a clade with GKaV and Hermitage virus (GenBank accession no. KU754512), a flavi-like virus identified in *Drosophila immigrans* (6). Whereas, a phylogenetic tree based on flavivirus helicase proteins placed DcFLV in a clade close to the *Wuhan centipede virus* (GenBank accession no. KR902737), identified in centipedes (*Otostigmus scaber* and *Scolopocryptops* sp.) (4). To our knowledge, this is the largest genome for a flavi-like virus identified to date.

### Accession number(s).

The GenBank accession number for DcFLV identified in this study is KX267823.

## References

[1] King AM, Carstens EB, Lefkowitz EJ (ed) 2012 Virus taxonomy. Classification and nomenclature of viruses. Ninth report of the International Committee on Taxonomy of Viruses. Elsevier Academic Press, San Diego, CA.

[2] Bekal S, Domier LL, Gonfa B, McCoppin NK, Lambert KN, Bhalerao K 2014 A novel flavivirus in the soybean cyst nematode. J Gen Virol 95:1272–1280. doi:10.1099/vir.0.060889-0. 24643877

[3] Kobayashi K, Atsumi G, Iwadate Y, Tomita R, Chiba K, Akasaka S, Nishihara M, Takahashi H, Yamaoka N, Nishiguchi M, Sekine K 2013 Gentian Kobu-sho-associated virus: a tentative, novel double-stranded RNA virus that is relevant to gentian Kobu-sho syndrome. J Gen Plant Pathol 79:56–63. doi:10.1007/s10327-012-0423-5.

[4] Shi M, Lin XD, Vasilakis N, Tian JH, Li CX, Chen LJ, Eastwood G, Diao XN, Chen MH, Chen X, Qin XC, Widen SG, Wood TG, Tesh RB, Xu J, Holmes EC, Zhang YZ 2016 Divergent viruses discovered in arthropods and vertebrates revise the evolutionary history of the *Flaviviridae* and related viruses. J Virol 90:659–669. doi:10.1128/JVI.02036-15. 26491167 PMC4702705

[5] Teixeira M, Sela N, Ng J, Casteel CL, Peng HC, Bekal S, Girke T, Ghanim M, Kaloshian I 2016 A novel virus from *Macrosiphum euphorbiae* with similarities to members of the family *Flaviviridae*. J Gen Virol 97:1261–1271. doi:10.1099/jgv.0.000414. 26822322

[6] Webster CL, Longdon B, Lewis SH, Obbard D 2016 Twenty five new viruses associated with the *Drosophilidae* (*Diptera*). Evol Bioinformatics 2:13–25. 10.4137/EBO.S39454 PMC4915790 27375356

[7] Grabherr MG, Haas BJ, Yassour M, Levin JZ, Thompson DA, Amit I, Adiconis X, Fan L, Raychowdhury R, Zeng Q, Chen Z, Mauceli E, Hacohen N, Gnirke A, Rhind N, di Palma F, Birren BW, Nusbaum C, Lindblad-Toh K, Friedman N, Regev A 2011 Full-length transcriptome assembly from RNA-Seq data without a reference genome. Nat Biotechnol 29:644–652. doi:10.1038/nbt.1883. 21572440 PMC3571712

[8] Li H, Durbin R 2010 Fast and accurate long-read alignment with Burrows-Wheeler transform. Bioinformatics 26:589–595. doi:10.1093/bioinformatics/btp698. 20080505 PMC2828108

